# Secondary School Students’ Reasoning About Science and Personhood

**DOI:** 10.1007/s11191-021-00199-x

**Published:** 2021-04-13

**Authors:** Berry Billingsley, Mehdi Nassaji

**Affiliations:** Faculty of Education, Canterbury Christ Church University, North Holmes Road, Canterbury, CT1 1QU Kent UK

## Abstract

Scientific advances, particularly in evolutionary biology, genetics, neuroscience and artificial intelligence, present many challenges to religious and popular notions of personhood. This paper reports the first large-scale study on students’ beliefs about the interactions between science and widely held beliefs about personhood. The paper presents findings from a questionnaire survey (*n* = 530) administered to English secondary school students (age 15–16) in which their beliefs and concepts regarding personhood and the position of science were investigated. The survey was motivated in part by an interview study and a previous, smaller survey which revealed that many students struggle to reconcile their beliefs with what they suppose science to say and also that some have reluctantly dismissed the soul as a ‘nice story’ which is incompatible with scientific facts. The results from this larger-scale survey indicate that a majority of the students believe in some form of soul. Even so, and regardless of whether or not they identified themselves as religious, most students expressed a belief that human persons cannot be fully explained scientifically, a position that some students perceived as a partial rejection of what it means to hold a scientific worldview.

## Introduction

The study reported in this paper sought to discover whether students perceive tension between what they suppose science to say and their beliefs about personhood and in particular the soul. The idea that each of us has soul is a cherished belief for many people but has become increasingly contentious as science has advanced. A Nobel Prize winner and biologist, Frances Crick, asserts that the astonishing conclusion of neuroscience is:

You, your joys and your sorrows, your memories and your ambitions, your sense of personal identity and free will, are in fact no more than the behaviour of a vast assembly of nerve cells and their associated molecules. As Lewis Carroll’s Alice might have phrased it: “you’re nothing but a pack of neurons” (Crick & Clark, 1994, p. 3).

The motivation for our study stems in part from the findings of an interview study in seven secondary schools in England to discover students’ perceptions of what science and religion say about the origins of life and the universe. This revealed that social and pedagogical pressures in science classrooms can make it difficult for students to explore connections between what they learn in science and what they learn in religious education, two subjects that are provided in all schools in England (Billingsley et al., 2016a, b). The findings prompted us to wonder whether the boundary that operates around what is discussed in the science classroom also prevents students from exploring more contemporary questions that are of interest to students with and without a religious faith. We hypothesised that this might be the case for beliefs about human personhood and ran a series of small-scale interview studies, focus groups and a literature review to investigate our hypothesis. The interviews provided many examples of students expressing uncertainty and sometimes discomfort about how they perceived science to interact with their beliefs about the person. In a later section of this paper, we will select and discuss some of quotations from those studies that drew our attention. These comments helped us to construct a series of statements that we incorporated into a questionnaire that was delivered to students in six secondary schools in England. The next paragraph explains further the rationale behind this survey in relation to the broader picture of our research.

## The School Curriculum

There is mounting pressure on schools internationally to do more to prepare students for the types of multidisciplinary questions they are likely to encounter as future citizens and scholars (OECD, 2018). Publically available descriptions of science have been brought into critical review by science educators as a result of media coverage of ministers’ speeches during the coronavirus pandemic (see, for example, Alsop & Bencze, 2020; Erduran, 2020; Levrini et al., 2020; Reiss, 2020) along with questions about whether science classrooms are adequately prepared to encourage criticality in their students. Erduran (2020, p. 488) writes in the editorial of a special edition of *Science and Education* the following:

Science education is vital in this mission and can potentially bring back nuance to how ‘science’ is characterised for science understanding. However, science education particularly school science in its conventional form, is unlikely to rise up to the challenge given that the complexity of the issues at hand require more than input from science. COVID-19 is not only a scientific problem. It is also a societal problem, appealing to politics and economics among other domains. A cross-curricular approach is needed where students can discuss a range of perspectives on science including how science may compare with other ways of knowing as well as what power and limitations science might possess.

One of the barriers that prevent this approach in England and elsewhere is a tendency to deliver the curriculum exclusively through discrete subject compartments each with its own objectives, teachers and resources (Paiva et al., 2019; Sheffield et al., 2019; Slomka, 2019). While this design successfully meets many objectives, it can prevent opportunities to see a question ‘top-down’ before it is apportioned to specialist disciplines. It means that students—and indeed adults—may develop a habit of keeping scientific and non-scientific statements in separate cognitive compartments and get little chance to see how each of these might inform their thinking about the other (Sadler, 2009; Wilthagen et al., 2018). The skills that are affected most are the ‘big picture’ thinking skills associated with technical and scientific literacy—the very thinking skills that all students need if they are to be wise and confident global citizens (OECD, 2018). Questions pertaining to personhood are in our view central and essential to this agenda, linking for example to public attitudes to organ donation (NHS, 2019), our relationships with other animals (Knight et al., 2003), artificial intelligence (Wakefield, 2016) restrictions on liberty during the COVID 19 pandemic (Pugh, 2020) and access to places of worship (Corbin, 2020). Questions about the nature of personhood are also important in the current context because adolescence is a key time for the development of personal identity (Kroger et al., 2010) and religious identity (Prawitasari, 2019) and also a time in life when questions about the nature of truth can seem paramount (Boyes & Chandler, 1992).

There is a basis to say that if science classrooms avoid discussing the strengths and limitations of science as a way to answer Big Questions about human personhood, science may be perceived by some students to take a scientistic stance on these issues. Scientism has been defined and characterised in a variety of ways (Dupré, 2001; Kidd, 2014; Peacocke, 1993; Sorell, 2013; Stenmark, 2013)). Bertrand Russell’s account of attainable knowledge is an explicit example of scientism and in our view, a useful example for students to examine. He states that ‘whatever knowledge is attainable, must be attained by scientific methods; and what science cannot discover, mankind cannot know’ (Russell, 1935). Critics of scientism regard it as no part of science, but instead understand it to be a philosophical, metaphysical, or ideological position (Feyerabend, 1988; Hutchinson, 2011). A series of survey and interview studies internationally and across several decades has found that a proportion of students perceive science in scientistic terms (Francis et al., 2019; Fulljames et al., 1991; Paiva et al., 2019; Pearce et al., 2019). We have previously stated our concern that in some cases, this perception of science may be adopted without examining other positions on the nature of science, a position we label as uncritical scientism (Authors, 2019). School students are encountering news of advances in evolutionary biology, neuroscience and genetics both in formal lessons and via the media. Many of the ideas presented have implications for widely held beliefs about personhood, for example, by suggesting that human thoughts, emotions and behaviours can to a greater or lesser extent be explained scientifically. Racine and colleagues (2010) have used the term ‘neuroessentialism’ to identify an emerging trend in media stories about neuroimaging to depict the brain as the essence of a person, with the brain a synonym for soul.

Concepts relating to life and personhood are discussed in most if not all science classrooms when a biology teacher gives a definition of life in terms of the characteristics of living things or discusses scientific findings in genetics that are pertinent to the notion of personhood. However, it seems reasonable to question whether as many biology teachers also explain that ‘what is life’ and ‘who I am’ are questions that can be addressed through a range of disciplines, each providing a different and possibly complementary perspective. To do so would help to ensure that students appreciate that scientism is not a necessary presupposition for science.

Thus, we formulated the following three research questions:

- RQ1: What are school students’ **own beliefs** about personhood, including how thinking and behaviour are determined, and whether humans have souls?
- RQ2: What do school students think **science says** in explaining human personhood including human thinking, behaviour and whether humans have souls?
- RQ3: To what extent school students perceive science and religion to be in conflict when explaining what it means to be a person?

## Development of the Questionnaire

Our aim was to prompt school students to think and talk about the relationships between science, religion, the wider humanities and their beliefs in the context of Big Questions. In the survey designed for this study, we wanted to discover whether and how students made sense of apparent contradictions between scientific and widely held non-scientific explanations. Conundrums are useful both as a research tool and as a stimulus during teaching because their puzzling nature can be highly motivating for students provided the puzzles are ones that students can relate to (Billingsley, 2004; White & Gunstone, 1992). The puzzles for this study were generated via two steps. The first was a literature review which sought to find examples in publications discussing the relationships between science and religion of tensions between science and widely held beliefs about personhood. This produced a set of themes that we present below.

### The Soul

The notion of a soul is central to our study and perspectives on soul frequently overlap with the other aspects of personhood that we are exploring. Brown (2004, p. 58) sets out some widely held attributes of the soul in a way that explains their relevance to personhood:

In many religious traditions, the concept of a soul has played a very important and meaningful role in the understanding of personhood. The soul has been thought to be the source of important aspects of human uniqueness, at various times including consciousness, intellect and free will. The soul is viewed as the point of interaction with God, and as necessary for maintaining belief in eternal life. It is the soul that is both corrupted by sin and the target of redemption. Most important the soul has come to encompass critical aspects of personhood (Brown, 2004, p. 58).

The notion of a soul as the essence and uniqueness of the person predates modern science. Keith Ward (2008) has reviewed what the six major world religions say about personhood. He concludes that five of the six include references to a soul. The Abrahamic religions (Judaism, Christianity and Islam) say that God created everything that exists and describe the soul as a conduit for expressing and experiencing a relationship with God. Sikhism and Hinduism both discuss the concept of soul to express a view on reincarnation. Sikhism, for example, says that at death the body is discarded like a garment while the soul moves on. These five religions refer to the soul as existing after death. Christianity refers to a time of resurrection when souls are re-embodied. Buddhism supports the notion of some kind of continuation for the psyche beyond death but denies the existence of individual souls. While the soul is a religious construct, it is also prevalent in popular culture and is frequently used in everyday language to convey the essence of what makes you who you are. It is widely believed that the soul (if it exists) is nonmaterial and separate to the rest of the person. Young people may encounter the idea of a separate soul that can leave the body in popular culture where authors depict what happens when life ends. In the children’s book, ‘*The Book Thief*’ by Markus Zusak (2005), Death is presented as a hooded character who stands invisibly by those who are dying, waiting to collect their soul. Zusak says that Death carries souls away from lifeless bodies ‘in my fingers like suitcases’ (p. 359). The idea of a soul as something that is separate to the rest of the person can be traced back to a philosophy conundrum rather than to a reading of religious texts.

The idea of a soul as the source of consciousness, conscience, free will and identity has become increasingly contentious as science has made advances in understanding the person. The background to this contention is complex because it involves not only the soul but also the mind. The reason for this conflation can be traced back to the rise of early modern science in the sixteenth and seventeenth centuries. At that time, in the West, scholarship was moving towards a new worldview which saw the universe as an elegant machine governed by exact mathematical laws (Capra, 1996). The philosopher and mathematician Descartes was impressed by the power of mathematics to model and predict the movements of material things. What was puzzling was why, since people are also made of matter, they seemed to act at will and get around these universal mathematical regularities. Descartes’ solution was to draw on Plato’s depiction of reality as divided into two realms—one material and one nonmaterial. Descartes applied this dualistic reasoning to say that a person has two parts, a material body and a nonmaterial ‘thinking thing … which doubts understands, affirms, denies, wills … and feels’ (Descartes & Veitch, 1960, p. 31). Gray (2010, p. 638) argues that the Bible does not promote the idea of a soul as ‘an extra separate spiritual thing inside a material shell’ but instead describes the person as ‘a living, breathing holistic entity’. The scientist and theologian John Polkinghorne (2013) and the Christian theologian Keith Ward (2008) also argue that the existence of a separate soul during life is not a necessary prerequisite for life after death.

John Polkinghorne (2004, p. 595) refers to a longstanding philosophical puzzle to propose that the soul is both ‘the real me’ and also ‘the almost infinitely complex information bearing pattern’ in which the matter of the body is organised:

Philosophers sometimes like to discuss the problem posed by a ship on a long voyage at sea. In the course of the journey, many piecemeal repairs prove necessary, to such an extent that when it reaches its destination there is not a plank or a mast that was actually part of the vessel when it set sail. Is it then the same ship that arrives in port, or is it a different one? I would say that it is the same ship if the pattern of its construction has been preserved.

Polkinghorne says that his position on the soul as a pattern that carries information explains why a person experiences life as one person, even though atoms change during a lifetime. Further, he says, it provides a reason to say that, via an act of God, a person can live again after death.

### Neuroscience and Genetics

Neuroscience and genetics are both reasons why some people might perceive science to take a scientistic stance. There are also many scholars who discuss the conundrum without taking one position or the other. McConnell and Philipchalk (1992, p. 6) argue that

We pay a price – and I think a heavy price – for framing the distinction between intentions and neurological activity in a deterministic way. If all intending is totally causally explained by neurological activity, our intentions do not have the significance we usually assign to them. They’re just an impotent add-on to what the brain was going to do anyway. I would further argue that the awareness that this is so may have undesirable consequences: If I believe that I am not ‘really’ guilty, because the attribution of guilt is not now based on an (illusory) ability to really choose, but is instead only an element in the armory of social engineering, I might be much less worried about acquiring guilt … However, these are grounds for exploring the possibility of finding an alternative account of human thinking: they are not by themselves adequate grounds for rejecting determinism. An uncomfortable truth is still a truth.

Advances in genetics are also changing our understanding of the role of genes in shaping humans’ emotion, thinking and behaviour. In particular, there is now widespread acceptance of epigenetic factors (factors that influence gene expressions) and these show how complex contextual factors may influence gene expressions (Watts, 2017).

### The Purpose of Life and Whether a Person Is Special

Our final two themes in the survey were whether life has a purpose and whether or not each of us is special and in some way intended to exist. We included statements to probe student attitudes to these ideas because they are beliefs about the person that are important to many religions and because they are dismissed by some people on the basis that they are perceived to conflict with evolutionary biology (see Alexander, 2008). There is a range of answers to the question of purpose in Biology. One reaction to the question might be denying any purpose and holding the idea that humans are just a happy accident. Others may respond from a religious point of view by maintaining ‘that the laws of the nature for life are entirely consistent with the older teleological religious concept of the cosmos as specially designed whole, with life and mankind as its primary goal and purpose’(Denton, 2002, xi). Similarly, the story from science of how we come to have attributes like intellect and self-reflection is evolution; this seems to contradict and replace the religious story of creation—which says we are special and intended. However, there are scientists who believe that human attributes are the outcome of evolution, but the evolutionary account does not explain away the religious account of human specialness (McGrath, 2020).

## Data Sourced from Interviews and Surveys

The second step was to review a very large data set gathered over time of students’ interview and survey comments to ascertain which if any of these themes were also addressed by students. During the review, we were also open to finding new points of tension and differing opinions by students on how to explain personhood. The survey data came from a survey which presented students with statements and invited students to express their level of agreement and add a comment if they wanted to (see Billingsley & Nassaji, 2019). The interview study was carried out with a sample of secondary school students (*n* = 25) in England aged 14–16 to discover whether they hold beliefs about personhood that they perceive to conflict with science (Authors, in review). The interview questions focussed on a number of interrelated concepts pertaining to personhood, such as the brain, the mind and the soul. Students were asked to critique each concept through a framework of what they supposed firstly science and then religion would say. They were also asked what they themselves believed on this topic and whether they perceived their belief to be consistent with science. In our report that follows, students’ real names have been replaced with pseudonyms.

Soul was the first topic we selected which resonated with the literature themes.

Kiera, aged 15, who took part in our interview study, said:

I think I have a soul. I think my soul is my conscience and, sometimes, my choices and, I think, if I was to die, I think people enjoy thinking that you’re going to live on through your soul and your conscience – that’s sort of where your personality comes from and is carried with you wherever you go. But, I think that’s quite romantic, isn’t it? [laughs] I think, a lot of what I believe, I believe because you don’t want to believe that you’re just going to – a soul is such a lovely idea and even though someone doesn’t have a soul – it all sounds so like a story book, you know. I’d like to think I have a soul, yeah.

Martha, explained:

I personally think the soul is [necessary to make a person] but I think that’s more of a religious thing. Scientists, I don’t think all of them believe that we have a soul because it’s nothing that can be proved, it’s something that’s spiritual, you can’t see it inside your body, but I personally think that it’s there.

Neuroscience and the relationship between the brain and the mind were the second theme we identified. How to explain human behaviour was the third. Relevant comments by students included:

Science will never be capable of reading minds.

If scientists look more in to the study of the brain they might be able to explain in full detail.

It is hard to tell how much science will progress in the distant future but I can’t see scientists being able to understand human behaviour fully in the next 20 years.

The final topics we identified were the purpose of life and being special, where comments by students included:

I believe that there is something more to human beings than just chemical reactions.

We are all different and this is what makes everyone individual and special.

At the end of our analysis of the survey comments and student interviews, we identified a set of six attributes of personhood which were frequently endorsed by students as relevant to their deliberations on personhood and the nature of truth. The six topics were as follows: (1) whether humans have souls; (2) the relationship between the brain and the mind; (3) the power of science in explaining human thinking; (4) the power of science in explaining human behaviour; (5) the purpose of life; and (6) whether each human person is special. The question of whether there is life after death is one that we did not raise explicitly because of the risk that it might cause a student distress if someone they love is very poor. When we constructed our survey for similar reasons, we chose to focus on seeking to understand how students perceive and explain the nature and attributes of a living person. Even so, it is a question that students sometimes raise themselves, and as such, we include it in the issues we discuss arising from the study.

## Piloting

Statements were piloted during an iteration of surveys with groups of students in the age range who came onto campus for workshops as part of an outreach programme. Students completed the surveys with different groups receiving different selections of statements and/or differently worded statements. Students indicated their level of agreement or disagreement with each statement and were also encouraged to provide a comment or explanation in the open box with each statement. Their responses informed further decisions about which statements to use and how they should be worded.

## Survey Statements

The survey included statements in each of the six categories of personhood we previously identified: soul, mind, thinking, behaviour, purpose and being special. For each statement about personhood, there is a statement of belief (corresponding to RQ1) and a statement about the perceived scientific position (corresponding to RQ2).

We also have a third research question and hence questions on the relationship between science and religion which addresses three categories for personhood—soul, behaviour and being special; there is also a fourth category: being human.

In table format, these statements are as follows (Table 1):

**Table 1:** List of statements and their relating topics and research questions

	The student’s belief	Perception of what science says	Relationship
Soul	I believe humans have souls	The scientific viewpoint is that the soul is not real	Science is compatible with religious ideas about the soul
Mind	The mind is the same as the brain Science can never fully explain the mind	Science says that once we understand how the brain works we will understand the mind	
Thinking	I believe that thinking will one day be completely explained by brain science	Science says our thoughts are determined by biological processes	
Behaviour	A person’s behaviour is something that science will never be able to fully explain One day science will be able to predict how a person will behave at every moment	Science says behaviour is determined by genes and upbringing	Scientific and religious explanations of human behaviour cannot both be right
Purpose	I believe that life has an ultimate purpose Science cannot tell us whether life has an ultimate purpose	Science says that there is no purpose to life other than the goal to survive	
Special	I believe that humans are special compared to other animals	Science says that humans are not special compared with other animals	Science and religion have conflicting views on whether humans are special
	There are aspects of being human that science cannot explore		Science and religion have conflicting views about what it means to be human

We acknowledge that some of the expressions linking science with scientism are less likely than others to stand out. The statements linking biological processes to how we think and behave both use the term ‘determined by’ rather than softer phrases such as ‘influenced by’ and the statement about human behaviour emphasises factors that are largely outside a person’s deliberate control—genes and upbringing. An interview is a more accurate way to gather students’ positions on science and scientism, however, our aim by designing and testing these surveys was twofold. Firstly we anticipated gaining more insight into the positions of the students taking part in the study. Secondly we wanted a set of statements that could be presented to students by teachers or facilitators in a workshop designed to increase their criticality and to ensure students meet a range of views. In that situation, the statements could also be given out before and after the lesson to assist with evaluation.

To look at whether students own beliefs are contradicted by science in the analysis, we can select their own beliefs and views of science from Table 2.

**Table 2:** List of statements used to compare student’s own belief and their views of science

Belief	Science view
I believe humans have souls	The scientific viewpoint is that the soul is not real
Science can never fully explain the mind	Science says that once we understand how the brain works we will understand the mind
One day thinking will be completely explained by science	Science says our thoughts are determined by biological processes
A person’s behaviour is something that science will never be able to fully explain	Science says behaviour is determined by genes and upbringing
I believe that life has an ultimate purpose	Science says that there is no purpose to life other than the goal to survive
I believe that humans are special compared to other animals	Science says that humans are not special compared with other animals

Once the statements were assembled, we added two more statements for positivity and hence balance, where science echoes the religious position. These were not analysed,

Science says that life has an ultimate purpose.

Science says that people choose how they behave.

We also included three statements that were used in a previous survey exploring attitudes to scientism. Two were already in the current survey and we added the third (which is about personality) in order to make comparisons between the samples in a future paper: One day science will be able to tell us how our personalities are formed.

The other two refer to behaviour and as such are already in the table above. They are the following: ‘A person’s behaviour is something that science will never be able to fully explain’ and ‘One day science will be able to predict how a person will behave at every moment’.

The survey also included some additional questions relating to categories we are not analysing in this paper to do with the relationship in general, interest, careers, experiences in the classroom and encounters with scientists: At the end of the survey, students were invited to state their gender and to choose between religious and non-religious positions. Students were able to skip any of the questions in the survey including these.

## Ethics and Sample

The survey was administered in six schools to students in year 10–11 (age 15–16) in six secondary schools in England. The schools were recruited via convenience sample using our wide range contact list of schools to seek a final group that were geographically diverse with some city, some rural, and a range of types and socioeconomic settings. Four are state-funded and two are privately funded, independent schools. Given the relevance of many of the survey questions to religion, a balance was sought between faith and non-faith schools, with three faith schools and three non-faith schools. Faith schools in England have a formal affiliation with a religious organisation but are required to teach a general curriculum. Most faith schools in England and all of the faith schools in our study are affiliated with the Church of England or the Catholic Church. Although not all state-funded schools follow the National Curriculum, government regulation prohibits the teaching of creationism as an evidence-based scientific theory (Long et al., 2019). Schools were provided with access to the survey online and to paper copies if they requested them. Students who participated in the study were selected by their teachers and completed the survey in school under supervision. Teachers were asked to choose an existing cohort such as a form group so that children did not self-select. We asked teachers to ensure that all the students were given a questionnaire. Students were told that their participation is voluntary and they could read the statements and not enter any responses if they chose. They could also skip any question they did not want to answer.

The questionnaire was completed by 530 students with 47% boys and 43% girls. A further 10% did not specify their gender. These students were included in the analysis of responses except where we compared the responses made by girls and boys.

Missing data was around 10% for most questions. A total of 426 of the pupils responded to the question ‘how would you describe your position on religion’, and of these, 41.3% selected Christian; 28.4% Atheist; 22.8% Agnostic; 4.7% Muslim; 1.4% Hindu; 0.9% Jewish; and 0.5% Sikh.

Table 3 provides more information about the sample.

**Table 3:** Composition of survey sample by school

Anonymised name	Type of school	Gender	Designated religious character	Percentage of children eligible for free school meals	Ofsted rating	Number of participants
PRS	Independent	Mixed	Non-faith	4%	No data available	205
STB	Academy convertor	Mixed	Faith (Church of England/Roman Catholic	15.3%	Good	66
STC	Academy convertor	Boys	Faith (Roman Catholic)	12.9%	Good	57
CHS	Academy convertor	Mixed	Non-faith	24.3%	Good	61
WEL	Independent	Mixed	Faith (Church of England)	0%	No data available	76
PSD	Girls academy convertor	Girls	Non-faith	4%	No data available	65

## Analysis

As the sample was large and each statement had five-level Likert items, we treated the data as interval data and assigned the following values to the five points: strongly agree = 1, agree = 2, neither agree nor disagree = 3, disagree = 4 and strongly disagree = 5 and reported the mean and standard deviation for each statement.

## Results and Discussion

### Students’ Positions on the Soul and Personhood

Just over half (53.0%) of those surveyed expressed belief that humans have souls. Interestingly, believing that the soul exists was not exclusive to religious students and about a third (34.3%) of respondents identified as non-religious strongly agreed or agreed with this statement. A similar proportion (51.2%) of the school students believed that life has an ultimate purpose while only 22.5% of them disagreed or strongly disagree with this belief. The level of agreement or strong agreement with the statement that humans are special compared with other animals was 42.3% and 31.8% of respondents disagreed or strongly disagreed with this statement.

As shown in Table 4, we found that the school students in our sample have the tendency to believe that humans are more than what science can explore. In particular, a majority of the school students in our sample believed that science can never fully explain the mind (57.0%). A majority of the school students (63.9%) strongly agreed or agreed that science cannot tell us whether life has an ultimate purpose. About a half (50.5%) of the school students believed that a person’s behaviour is something that science will never be able to fully explain. With regard to the general view of what it means to be human and whether there are aspects of being human that are beyond the scope of science, 58.5% strongly agreed or agreed with the statement that ‘there are aspects of being human that science cannot explore’ while only a fifth (20.7%) disagreed or strongly disagreed with the statement; just over a half (53.7%) of respondents who strongly agreed or agreed with this statement positioned themselves as religious while just under a half (46.3%) positioned themselves as non-religious.

**Table 4:** Students’ positions on the soul and personhood

	Strongly agree	Agree	Neither agree nor disagree	Disagree strongly disagree	Strongly Disagree	Mean	SD
Statements about beliefs that resonate with religious ideas							
I believe humans have souls	21.5%	31.3%	23.3%	12.7%	10.2%	2.56	1.207
I believe that humans are special compared with other animals	10.5%	31.8%	23.1%	22.4%	9.4%	2.80	1.169
I believe life has an ultimate purpose	16.6%	34.6%	25.5%	14.4%	8.1%	2.60	1.152
Statements that there is more to being human than science can say:							
A person’s behaviour is something that science will never be able to fully explain	14.9%	35.6%	22.9%	19.2%	7.0%	2.66	1.157
Science can never fully explain the mind	18.7%	38.3%	23.2%	15.7%	4.1	2.48	1.088
Science cannot tell us whether life has an ultimate purpose	13.9%	50.0%	24.1%	8.5%	3.4	2.37	0.943
There are aspects of being human that science cannot explore	21.8%	36.7%	22.7%	12.9%	7.8%	2.54	1.098
Statements that science fully explains the person							
One day science will be able to predict how a person will behave at every moment	4.5%	15.5%	25.4%	34.5%	20	3.50	1.111
The mind is the same as the brain	7.4%	28.6%	22.4%	33.2%	7.1%	3.00	1.092
I believe that thinking will one day be explained by brain science	10.2%	32.1%	28.5%	23.1%	6.2%	2.83	1.086

Conversely and consistently with these beliefs, in regard to statements that science fully explains the person: just over a third (36.0%) of the school students in our sample believed that the mind is the same as the brain while a higher proportion (40.3%) disagreed or strongly disagreed and about a quarter (22.4%) chose the ‘neither agree nor disagree’ option. The majority of the school students (57.8%) did not believe that thinking will one day be completely explained by brain science. Over half of the school students believed that it is not the case that one day science will be able to predict how a person will behave at every moment (54.5%).

### Students’ Perceptions of What Science Says About Human Personhood, and Whether Science Dismisses the Idea of Soul

We turn next to the statements that asked students to give the position science takes on the soul and other aspects of personhood. We found that 40.5% of the school students in the sample agreed or strongly agreed with the statement that the scientific view is that the soul is not real. About a third (35.3%) of respondents agreed or strongly agreed with the statement that ‘science says that once we understand how the brain works we will understand the mind’. More than a half (57.9%) of the respondents strongly agreed or agreed with the statement that ‘science says behaviour is determined by genes and upbringing’. A similar proportion (56.6%) agreed or strongly agreed with the statement that ‘science says our thoughts are determined by biological processes’. Roughly equal proportions of the school students agreed or strongly agreed (34.9%), neither agreed nor disagreed (28.9%) and disagreed or strongly disagreed (36.2%) that science says that there is no purpose in life other than the goal to survive. Likewise, similar proportions strongly agree or agree that ‘science says that humans are not special compared to other animals’ (34.1%) as disagreed or strongly disagree (32.4%).

In this sample, school students’ positions on the power of science in explaining human personality (responses to the statement: there are aspects of being human that science cannot explore) are almost equally distributed into three categories of strongly agreed or agree, neither agree nor disagree and disagree or strongly disagree.

**Fig. 1:**
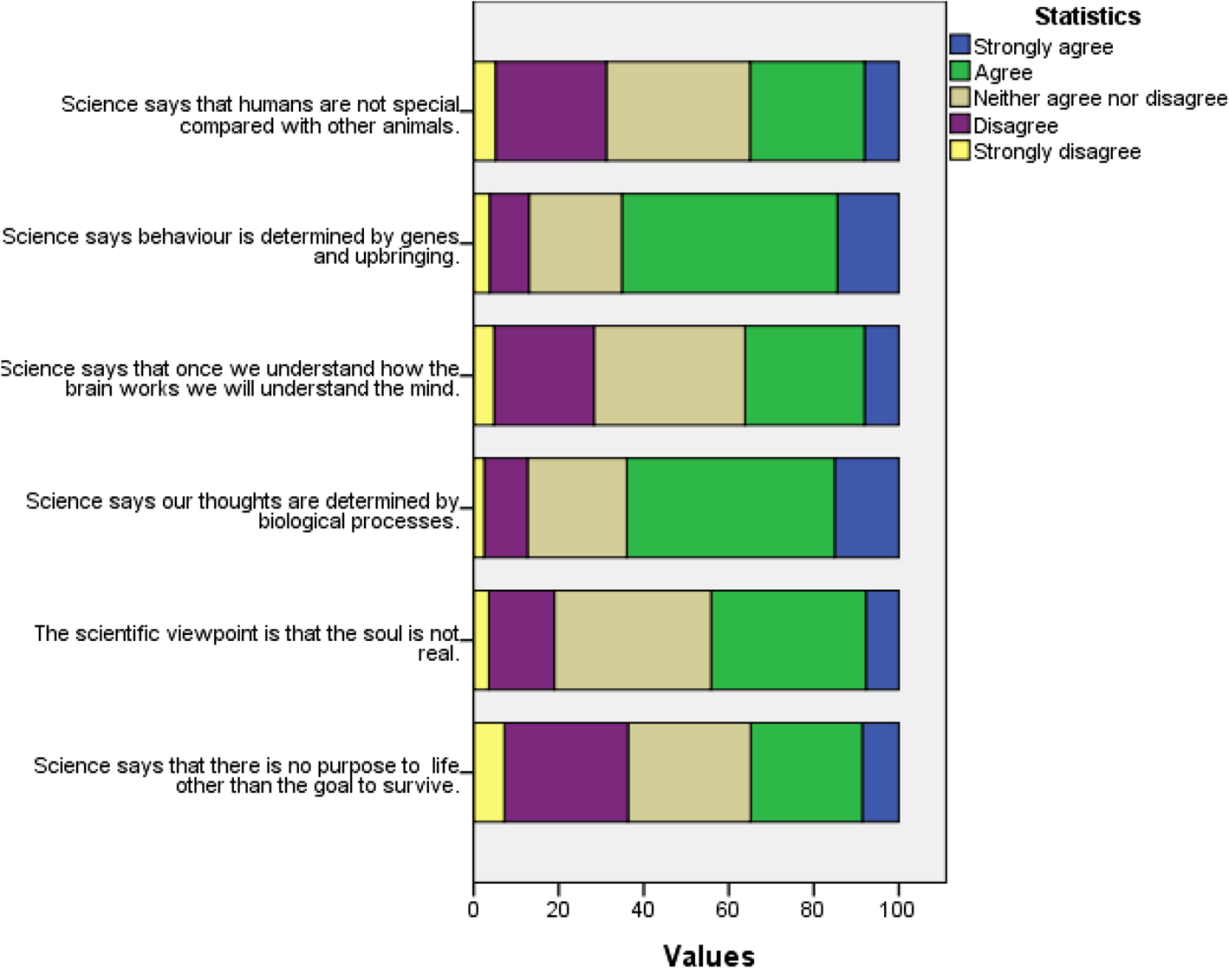
Stacked bar chart for statements relating to students’ views of science in explaining human personhood, and whether science dismisses the idea of soul

Figure 1 and Table 5 show that while the school students vary in their beliefs about the power and limitations of science, there is an inclination in our sample to accept statements identifying science as scientistic. There are several ways to explain this finding. There are likely to be students in this group who hold considered stances on the nature of science and who perceive science to have the power to fully explain human personhood. The group may also include some students who are not critically examining the stance expressed in these statements about the nature of science when they indicate agreement.

**Table 5:** Students’ views of science in explaining human personhood, and their views of the position of science on whether the soul exists

	Strongly agree	Agree	Neither agree nor disagree	Disagree	Strongly disagree	Mean	SD
Science says that humans are not special compared to other animals	7.2%	26.9%	32.9%	27.6%	5.3%	2.96	1.008
Science says behaviour is determined by genes and upbringing	14.1%	43.8%	22.1%	14.6%	5.4%	2.53	0.944
Science says that once we understand how the brain works we will understand the mind	8.7%	26.6%	35.1%	25.2%	4.5%	2.90	1.011
Science says our thoughts are determined by biological processes	13.8%	42.8%	23.9%	15.4%	4.1%	2.53	0.937
The scientific viewpoint is that the soul is not real	7.5%	33%	36.4%	18.4%	4.7%	2.79	0.934
Science says that there is no purpose in life other than the goal to survive	8.6	26.3	28.9	29.1	7.1	3.00	1.090

### Extent to Which Students’ Own Beliefs Conflict with Their Beliefs About Science

Just over a third (34.4%) of those who believed that humans have souls also strongly agreed or agreed with the statement that ‘the scientific view is that soul is not real’ indicating they perceive science to take a position that conflicts with their own.

Among those students in our sample who strongly agreed or agreed that ‘science can never fully explain the mind’, 28.7% strongly agreed or agreed that ‘science says that once we understand how the brain works we will understand the mind’—indicating that they perceive science to hold a position that conflicts with their own.

Out of those students who strongly agreed or agreed that ‘a person’s behaviour is something that science will never be able to fully explain’, more than half (57.1%) strongly agreed or agreed that ‘science says behaviour is determined by genes and upbringing’.

If we look at the group of respondents who disagreed or strongly disagreed with the statement that one day thinking will be completely explained by science, 54.5% of them strongly agreed or agreed that ‘science says our thoughts are determined by biological processes’ indicating that they perceive science to take a position conflicting with their own.

Our analysis revealed that 17% of respondents personally believe that humans have souls and at the same time believe that science says that the soul is not real. Near to 13% believe that the science can never fully explain the mind while attributing the view that once we understand the brain we will understand the mind to science. Twenty eight percent of the respondents in our sample strongly agreed or agreed with the statement that ‘a person’s behaviour is something that science will never be able to fully explain’ and at the same time believed that ‘science says behaviour is determined by genes and upbringing’. Sixteen percent of the whole cohort strongly agreed or agreed with the statement that I believe that thinking will one day be completely explained by brain science, and at the same time believe that science says that thoughts are determined by biological processes. Fifteen percent of the respondents expressed a belief that life has an ultimate purpose while believing that science says that there is no purpose to life other than to survive, and 12% believed that humans are special compared to other animals, while believing that science disagrees.

Averaged across the six topics explored in this study (soul, mind, behaviour and thinking, purpose of life and being special), 15% of the sample found an explicit conflict between what they believe and what they perceive science to say. By the term ‘explicit conflict’, we mean that they have identified a personal position on a topic and have also identified what they perceive as the scientific view is on the topic and these two positions are incompatible. Moreover, our analysis shows that 56% of participants found an explicit conflict on at least one topic (among six topics) between their own beliefs and what they think science says (see Table 6).

**Table 6:** Extent to which students’ own beliefs are in conflict with science

Theme	Existence of the soul	Reduction of the mind to brain	How behaviour is explained	How thinking is explained	Life having a purpose	Humans being special
Percentage of perceived conflict between own belief and knowing what science says	17%	13%	28%	16%	15%	12%

56% of participants found an explicit conflict on at least one topic (among six topics) between their own beliefs and what they think science says

### Conflict Between Science and Religion in Explaining Personhood

**Fig. 2:**
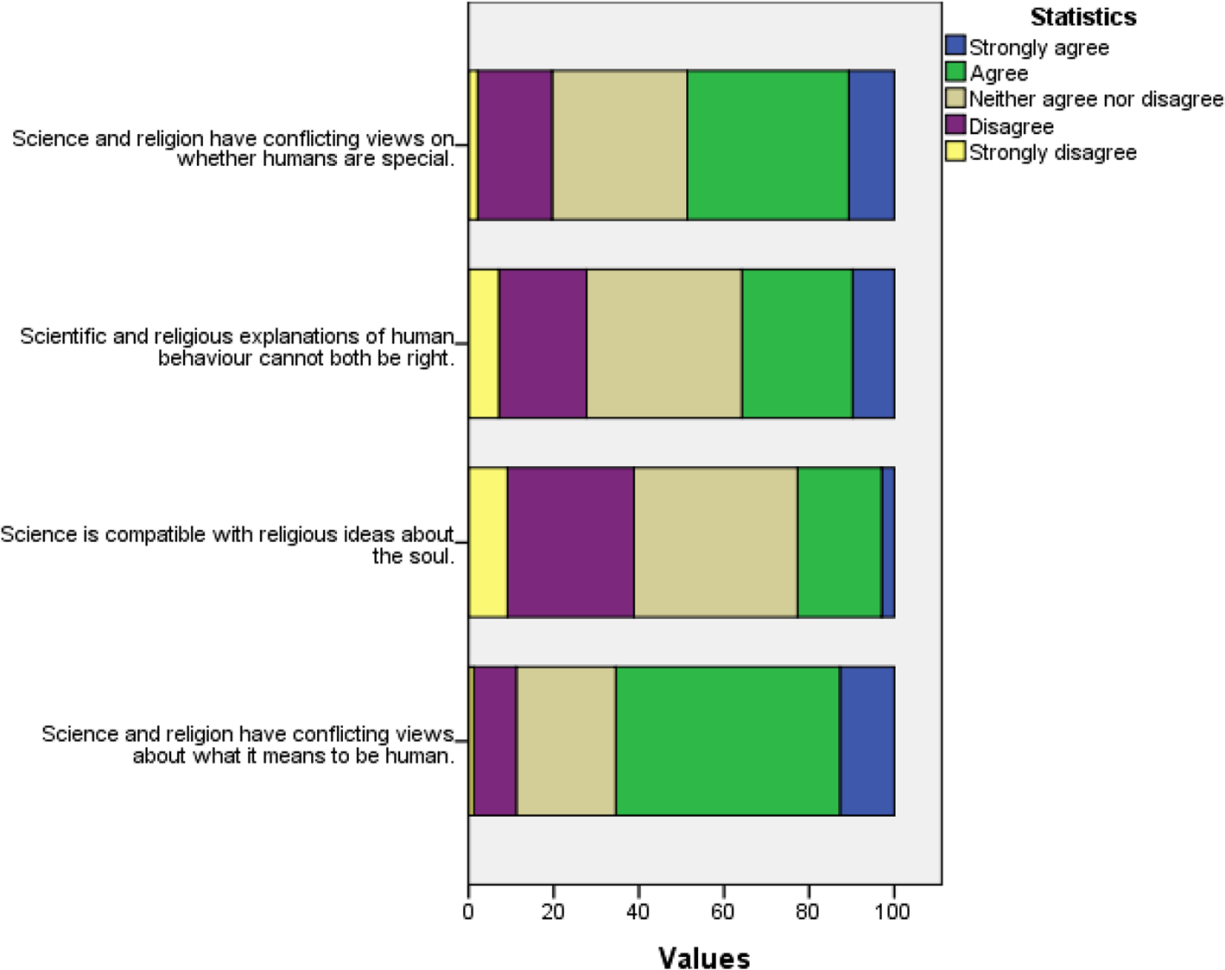
Stacked bar chart for statements relating to perceiving conflict between science and religion in explaining personhood

So far, we have looked for tensions between school students’ own beliefs about personhood and what they perceive science says. In this section, we report on the statements that are designed to discover perceived conflicts between religion and science in explaining personhood. With regard to the concept of the soul, 27.1% believed that science and religion have compatible views, 33.4% disagreed or strongly disagreed with this position and about 39.4% chose the ‘neither agree nor disagree’ option (Fig. 2). With regard to the explanation of human behaviour, 36.2% stated that scientific and religious explanations cannot both be right. 42.6% of the respondents believed that science and religion have conflicting views on whether humans are special and 24.2% of the respondents disagreed or strongly disagreed with this statement. In addition, 57.4% strongly agreed or agreed that science and religion have conflicting views about what it means to be human (Table 7).

**Table 7:** Conflict between science and religion in explaining personhood

	Strongly agree	Agree	Neither agree nor disagree	Disagree	Strongly disagree	Mean	SD
Science and religion have conflicting views about what it means to be human	11.0%	46.4%	24.1%	15.7%	2.8%	2.52	0.890
Science is compatible with religious ideas about the soul	3.6%	23.5%	39.5%	26.3%	7.1%	3.09%	0.956
Science and religion have conflicting views on whether humans are special	9.4%	33.2%	33.1%	21.2%	3.0%	2.74%	0.968
Scientific and religious explanations of human behaviour cannot both be right	10.5%	25.7%	35.0%	22.1%	6.7%	2.88%	1.050

It is interesting to see how many students chose the middle option, ‘neither agree nor disagree’, for the questions pertaining to personhood. For example, when asked whether scientific and religious explanations of human behaviour cannot both be right, just over a third (35.0%) selected neither agree nor disagree, while 33.1% concerning whether science and religion have conflicting views about whether humans are special, and 39.5% concerning whether science is compatible with religious ideas about the soul. When we returned to our qualitative data from survey comments and interview studies, looking for a possible explanation, we were reminded that students’ lived experiences and reflections on their own behaviour seemed to suggest a third option to many of them, which is that behaviour and thinking are changeable, spontaneous or random. Here are some examples:

The brain can be unexpected, obviously there are correlations of what people might do but never will they be able to fully understand behaviour.

Some people’s behaviours do not fit into the regular pattern.

It can change for the person, mood swings and things like that.

Apart from the main question we addressed in this study, there are some other research questions that we explored, prompted by the data available to us. We were interested to know whether there is a difference between faith and non-faith schools, and also girls and boys in their belief about being human and what they think science says about personhood.

#### Differences Between Girls and Boys

As we had no directional hypotheses about gender differences, we employed two-sided *t*-tests, and selected Welch’s test as this is more robust in cases of non-normally distributed data. Independent sample *t*-test (Delacre et al., 2017). In general, the girls in the sample were more inclined to think that there is more to being human than what science can investigate, though in some cases the differences were not statistically significant. For instance, girls strongly agreed or agreed less than boys with the statement that ‘the mind is the same as the brain’ (*t* = 2.96, df = 470, *p* = 0.003). Girls also strongly agreed or agreed less with the statement ‘one day science will be able to tell us how our personalities are formed’ (*t* = 1.191, df = 456, *p* = 0.047). The statement ‘there are aspects of being human that science cannot explore’ was more favoured by girls (*t* = 2.254, df = 448, *p* = 0.025) and they believed in the soul more than boys (*t* = 3.413, df = 462, *p* = 0.001).

#### Differences Between Faith and Non-faith Schools

There is no clear picture as a result of making comparisons between faith and non-faith schools in our sample. As before, we used two-tailed Welch’s independent sample *t*-tests to examine differences between these groups.

We found that the students from faith schools expressed higher agreement with the statement ‘I believe humans have souls’ compared to students from non-faith schools (*t* = 2.878, df = 505, *p* = 0.004). Similarly, the students from faith schools were more likely to agree that humans were special compared to other animals compared to students from non-faith schools (*t* = 3.679, df = 499, *p* = 0.001).

However, the picture is mixed as conversely students from faith schools in our sample are more likely to agree that ‘the mind is the same as the brain’ compared to students from non-faith schools (*t* = 2.911, df = 505, *p* = 0.006). They were also more likely to agree that ‘one day science will be able to predict how a person will behave at every moment’ (*t* = 2.435, df = 560, *p* = 0.015).

## Conclusion

In this study, we drew on our previous strategy of using conundrums to prompt students to talk about the relationships between science and non-scientific beliefs to design a survey which we then used to find out about students’ understanding of human personhood. We proposed this theme of personhood on the basis that we surmised it would interest a significant proportion of students. There is a lack of agreement even among religious people about what the soul is like. This means that scholars entering the debate choose which version they argue for or against. Traditional science courses focus on raising children’s understanding of well-established science concepts and in-discipline relationships. Ratcliffe (2009) made the point that if the focus in a lesson is a big question that bridges curriculum areas, it is key to have a clear set of assessable outcomes as without these, the lesson can drift into amorphous discussion. Recognising the importance of this point, we posit that the statements in this questionnaire could be used in a classroom setting to encourage critical thinking about the nature of science and, where appropriate, to broaden students’ understanding of the range of views that exist—about science and the soul. There is also the question of where teaching about Big Questions should take place and in which curriculum subjects (Ratcliffe et al., 2005). We recommend placing lessons that ask questions across discipline in a multidisciplinary space such as a library. Doing so helps to establish for students the idea that we can ‘move into’ the mind-set of an individual discipline by going into its specialist room and also come together with scholars across disciplines to tackle big questions and also to continue to learn what makes each specialism distinctive compared with other areas of knowledge. The research and findings described in this paper indicate that there are likely to be students who would benefit from a supervised exploration and chance for questions and discussion. The survey found that a proportion of those in our sample felt there to be some degree of conflict between their own beliefs and what they believed science says about personhood. For example 17% of the sample said that they believe in a soul and at the same time perceived that science says that the soul is not real. We also found that 56% of respondents perceived a conflict on at least one of the six topics. There were high levels of agreement with the statements that ‘science says behaviour is determined by genes and upbringing’ and ‘science says our thoughts are determined by biological processes’. These statements were designed to suggest that in a scientific view, personality and thinking can be explained using factors outside conscious control. These and other statements in the questionnaire could be useful in a lesson about the nature of science. Students could be invited to critique the wording and change it if they so wished to reflect their own positions.

## Recommendations for Curriculum Development

Earlier we made a case for the importance of preparing students for a future in which science plays a hugely significant role—but not an exclusive role—in the key questions and issues facing individuals and society. In a specialist classroom, teachers may find themselves taking shortcuts with the language they use in explanations. In a science classroom, there may not seem to be a reason to qualify in each lesson what we mean by scientific evidence in comparison with historical and other perspectives on what evidence includes. However, a significant proportion of students may not have thought deeply about the nature of knowledge and may not understand how science is similar and different to other ways of thinking. This means that the curriculum has a role to play in calling on teachers to emphasise key ideas about the nature of knowledge regularly, so that the messages are introduced and reinforced.

**Fig. 3:**
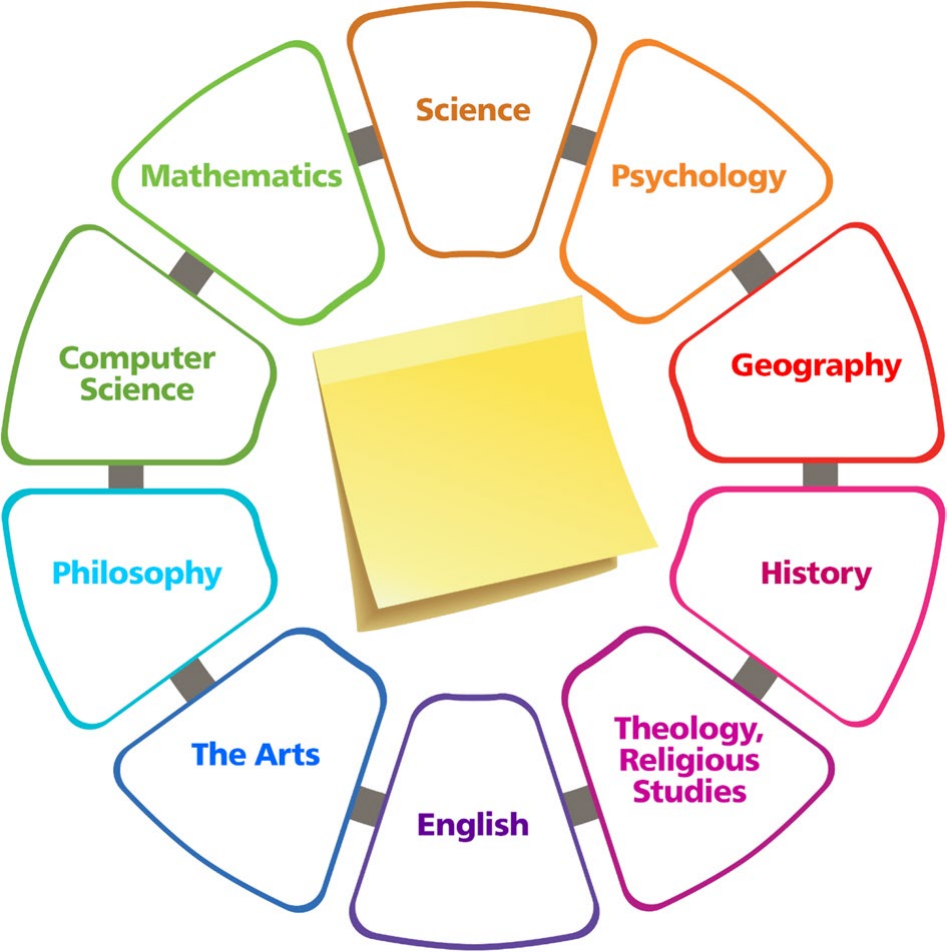
The Discipline Wheel

Secondly developing young people’s epistemic insight into the nature of knowledge goes hand in hand with developing their curiosity about the Big Questions themselves. Big Questions are frequently metaphysically sensitive, which is to say that scholars do not agree about the extent to which science alone can resolve them. Encountering and comparing scholars with different views on this issue can give students sight of questions and scholarship in the context of a debate which of itself can add to its potential to engage. The concept of emergence and non-reductive explanations of personhood underpin many contemporary explanations of soul and conscience (Murphy, 2016; Polkinghorne, 2013). A philosopher, Mary Midgley (2013, 2014, 2018) argued that there are different levels of explanation, which we study with different tools and in different contexts. We noted previously that in school, science and other (curriculum) disciplines in schools are presented in a school timetable as separate and haphazardly arranged boxes. We would like to see a curriculum that specifies the addition of some lessons to explore personhood ‘in the round’ using tools like the Discipline Wheel (see Fig. 3). These questions, such as whether or not humanoid robots can or should ever be conferred the status of persons, and the extent which we create our own futures, are important questions for individuals and society. We recommend designing, running and testing activities and workshops to increase students’ familiarity with science and technology and raise their epistemic insight into the power and limitations of science. The intended learning outcomes are to help school students to be able to recognise and distinguish questions that are amenable to science and questions that are more metaphysically sensitive, as well as to recognise that scholars differ in the extent to which they believe these different kinds of questions can be addressed through scientific research alone. Through further research, we will seek to discover if this and other strategies are effective ways that secondary school teachers can deliver their separate specialisms while also supporting students with how to ask and address big questions that span science, religion and the wider humanities.

## Recommendations for Broader Education Policy

To make progress with questions of human personhood and the nature of reality—we need to have insight into the ways of our own thinking and of particular relevance here, to identify and reflect on the strengths and weaknesses of the education system that has brought each of us to this point. Education over the world has adopted similar patterns and structures and so any impacts of systemic pedagogies and organisational structures are likely to be widespread and deep. Our research also reveals that Big Questions and multidisciplinary ways of thinking are seen by many students and many girls in particular as relevant and important in their lives. We ‘miss a trick’ as science educators if we avoid these links or if we hint that our subjects can address Big Questions but do not follow through with the details of what this engagement looks like. Scholars who are part of the science-religion dialogue become practised and comfortable with talking critically about the nature of knowledge and have developed language tools and thinking habits that serve their purposes. In this research, we have identified and investigated a concern that the structure of education can mean that it is difficult for students to have access to that language and those ideas. Addressing this gap where it exists seems to us to be a way to increase student agency and criticality while modelling the value of a life-long interest in asking and exploring Big Questions that are puzzling them.
